# Trion Transfer in Mixed-Dimensional Heterostructures

**DOI:** 10.1021/acsnano.5c17799

**Published:** 2026-04-02

**Authors:** Nan Fang, Ufuk Erkiliç, Yih-Ren Chang, Shun Fujii, Daiki Yamashita, Chee Fai Fong, Satoru Morito, Kaito Kanahashi, Takashi Taniguchi, Kenji Watanabe, Keiji Ueno, Kosuke Nagashio, Yuichiro K. Kato

**Affiliations:** † Nanoscale Quantum Photonics Laboratory, 13593 RIKEN Pioneering Research Institute, Saitama 351-0198, Japan; ‡ Quantum Optoelectronics Research Team, 222788 RIKEN Center for Advanced Photonics, Saitama 351-0198, Japan; § Department of Physics, Keio University, Yokohama 223-8522, Japan; ∥ Photonics-Electronics Integration Research Center, National Institute of Advanced Industrial Science and Technology (AIST), Tsukuba, Ibaraki 305-8568, Japan; ⊥ Department of Chemistry, 13032 Saitama University, Saitama 338-8570, Japan; # Department of Materials Engineering, 13143 The University of Tokyo, Tokyo 113-8656, Japan; ∇ Research Center for Materials Nanoarchitectonics (MANA), National Institute for Materials Science, Tsukuba, Ibaraki 305-0044, Japan; ○ Research Center for Electronic and Optical Materials, 52747 National Institute for Materials Science, Tsukuba, Ibaraki 305-0044, Japan

**Keywords:** carbon nanotubes, tungsten diselenide, heterostructures, photoluminescence, trion transfer

## Abstract

Charged excitons, or trions, offering spin and charge degrees of freedom, have primarily been investigated in doped systems where charges are long considered indispensable. Here, we present an alternative route to ultraefficient trion emission from an intrinsic, defect-free semiconductor via a transfer mechanism. By exciting trions in two-dimensional tungsten-diselenide donors and transferring them into one-dimensional carbon-nanotube acceptors in mixed-dimensional heterostructures, we circumvent the usual carrier requirement, overcoming intrinsic Auger-quenching limitations. Benefiting from a reservoir effect induced by dimensional heterogeneity, this process achieves trion emission efficiencies increased by over 100-fold compared to conventional doping-based approaches, and remains robust across diverse doping conditions. Our findings extend the exciton-transfer paradigm to the three-body quasiparticles, offering a platform for advancing excitonic physics and trion-based optoelectronic/spintronic applications.

## Graphical abstract

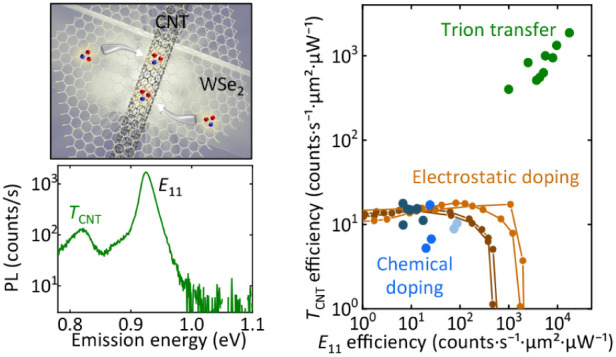

Excitonic processes lie at the heart of modern optics, photonics, and optoelectronics.^1–4^ The transfer of excitons between dissimilar materials, where photoexcitation in one material leads to emission in another, is a fundamental phenomenon observed in organic semiconductors,^5^ quantum dots,^6^ carbon nanotubes (CNTs),^7,8^ transition metal dichalcogenides (TMDCs),^9^ and other excitonic systems.^10,11^ Harnessing such exciton transfer can expand the absorption spectrum,^6,8,9^ boost quantum yields,^12^ and enable novel device concepts for light-emitting diodes,^13^ solar cells,^14^ biosensing,^15^ and other energy-harvesting applications.^16^

Another intriguing class of quasi-particles is the trion, a charged exciton formed by binding an exciton to an additional electron or hole. Trions carry net charge and spin, making them appealing for spintronics and quantum information technology.^17–19^ Since the early observations in 1993 within doped quantum wells,^20^ trions have almost always been studied in doped materials.^21–23^ Carriers are presumed essential for trion formation, which is typically supplied by electrostatic gating, chemical doping, or impurity doping. Besides the carriers that bind with excitons to form trions, the excess charges inevitably impact nearly every trion property. These charges limit trion emission through strong nonradiative Auger recombination^24,25^ and introduce many-body complexities that often necessitate describing trions within the exciton-polaron framework.^26,27^ Generating a “pure” trion flux in a charge- and trap-free emitter, which would avoid the above interactions and enable potential trion-based spin qubits, has remained elusive under the existing paradigm.

Here, we introduce a fundamentally new concept—trion transfer—to achieve efficient trion emission from an intrinsically neutral, defect-free emitter. It is realized in a mixed-dimensional heterostructure consisting of one-dimensional (1D) CNT and two-dimensional (2D) tungsten diselenide (WSe_2_). Rather than doping the CNT, photoexcited trions in the 2D donor are transferred to the 1D acceptor, circumventing the usual nonradiative limitation imposed by free carriers. Through photoluminescence excitation, spatial imaging, and time-resolved measurements, we unveil a pronounced “trion reservoir” effect arising from dimensional heterogeneity, achieving emission efficiencies exceeding those of conventional doping-based methods by over 2 orders of magnitude. In a field-effect transistor configuration where the CNT is tuned from neutral to highly doped state, the transfer-induced trion emission remains strong, distinguishing this free-carrier-insensitive mechanism from conventional optical processes. Moreover, by doping the WSe_2_ donor with Nb, we show that trion transfer preserves high efficiency even in less-ordered systems where charge transfer coexists. These findings extend the fundamental understanding of the quasiparticle transfer process from excitons to trions, highlighting its unique optical route for achieving “pure” trion formation. A dense trion flux confined to a 1D channel opens new avenues in trion physics, spintronics, and advanced optoelectronic applications, for example the use of distinctive Fermi pressure absent in neutral excitons and the realization of trion superfluorescence.

## Results and Discussion

### Trion Emission in Mixed-Dimensional Heterostructures

We construct the CNT/WSe_2_ heterostructures by transferring a WSe_2_ flake onto an individual air-suspended CNT using the anthracene-assisted technique.^7,8^ The positions and chiralities of the CNTs are identified by photoluminescence (PL) spectroscopy^32^ and the layer number of WSe_2_ is determined before transfer, ensuring a clean and well-defined 1D/2D interface (Figure 1a,b). Optical images confirm that all studied samples possess a large, uniform suspended WSe_2_ region exceeding 5 μm in length that fully covers the CNTs. Notably, the CNTs in this study are defect-free and undoped as characterized by PL measurements,^33^ whereas the natural WSe_2_ crystals exhibit slight and unintentional doping due to intrinsic defects^34^ as evidenced by the presence of the trion peak in the PL spectrum of a suspended monolayer WSe_2_ flake (Figure S1).

**Figure 1:**
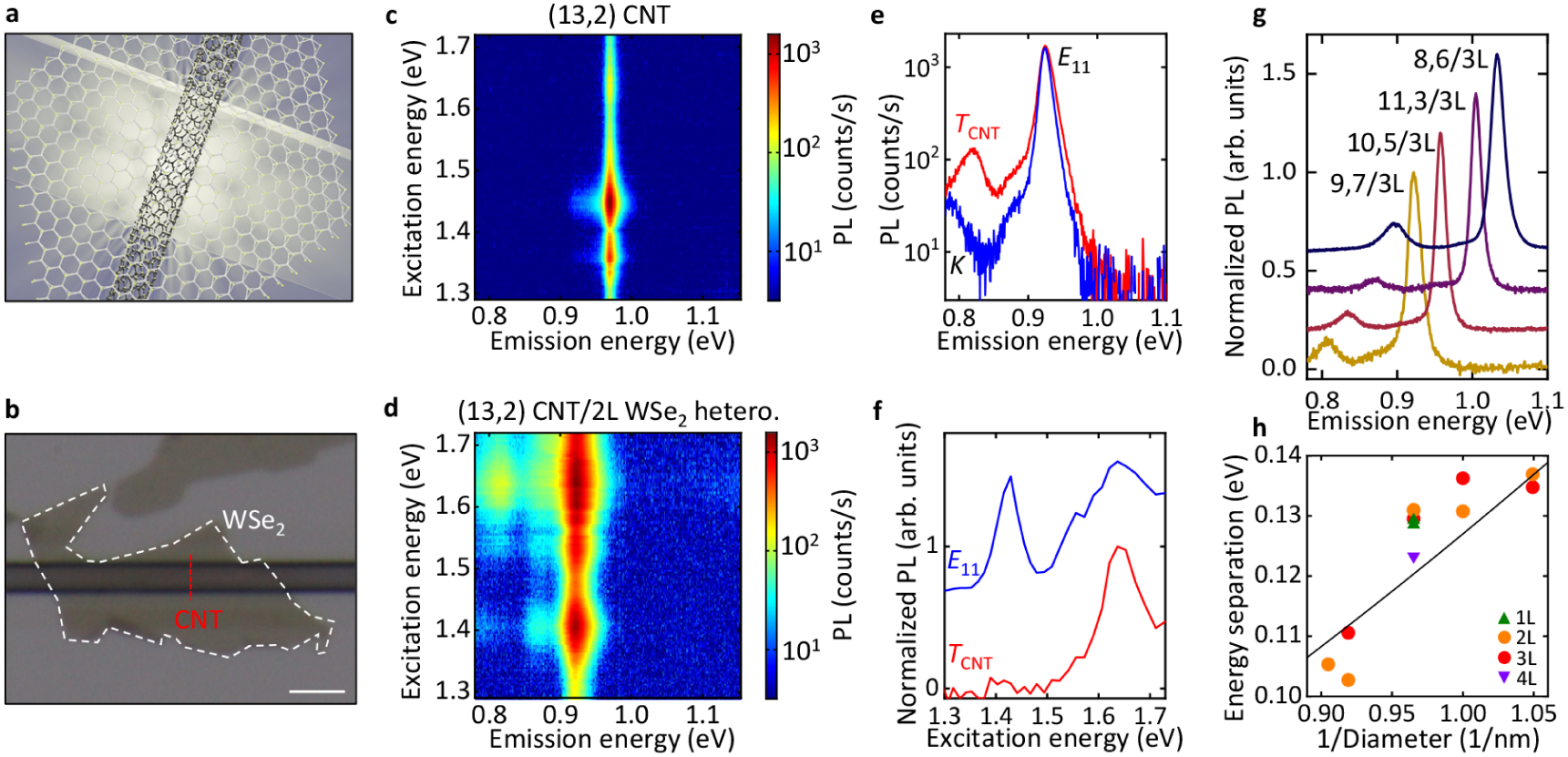
(a) A schematic of a suspended CNT/WSe_2_ heterostructure. (b) A typical optical image of a CNT/2L WSe_2_ sample. The CNT is indicated by the broken red line. The scale bar represents 3 μm. (c) The PLE map of the pristine (13,2) CNT. The excitation peaks at 1.640 and 1.359 eV correspond to the 2*u* excited exciton state^28–30^ and the phonon sideband of the *E*_22_ exciton,^31^ respectively. (d) The PLE map of the (13,2) CNT/2L WSe_2_ heterostructure, where the tube differs from that in (c). (e) The PL spectra of the (13,2) CNT/2L WSe_2_ heterostructure in (d) at excitation under *E*_22_ (blue, 1.420 eV) and $T_{{\mathrm{WSe}}_{2}}/X_{{\mathrm{WSe}}_{2}}$ (red, 1.634 eV), respectively. (f) Normalized PLE spectra of integrated *E*_11_ emission (blue) and *T*_CNT_ emission (red) in (d). The PL emission is integrated over a 10 meV-wide spectral window centered at the *E*_11_ or *T*_CNT_ energies. (g) Chirality-dependent PL spectra at $T_{{\mathrm{WSe}}_{2}}/X_{{\mathrm{WSe}}_{2}}$ excitation. We define a heterostructure nomenclature where (9,7) CNT/3L WSe_2_ is represented by (9,7)/3L. (h) Energy separation as a function of 1/Diameter from different samples. Green, orange, red, and purple symbols represent the heterostructures with 1L, 2L, 3L, and 4L WSe_2_, respectively. The excitation power is 10 μW. The black line is a fit.

Photoluminescence excitation (PLE) measurements provide direct insight into trion emission in these heterostructures. As a representative example, Figure 1c shows a PLE map of a pristine (13,2) CNT, where the *E*_22_ (1.447 eV) and *E*_11_ (0.971 eV) transitions are clearly observed. In a (13,2) CNT/bilayer (2L) WSe_2_ heterostructure (Figure 1d), these peaks redshift to 1.408 and 0.923 eV, respectively, due to the dielectric screening by WSe_2_. The bright *E*_11_ emission of the heterostructure suggests that the CNT remains largely undoped. Additionally, a new excitation peak at 1.643 eV arises from exciton transfer of the WSe_2_ A excitons $\left(X_{{\mathrm{WSe}}_{2}}\right)$ to the CNT *E*_11_ excitons, denoted as $\left|X_{{\mathrm{WSe}}_{2}}\right\rangle \to \left|E_{11}\right\rangle$ , consistent with transfer via tunneling for a type-I band alignment.^8^

When excited near the $X_{{\mathrm{WSe}}_{2}}$ peak, a prominent low-energy emission peak emerges at 0.817 eV as shown in Figure 1d. The energy separation Δ*E* between this subpeak and *E*_11_ is 0.106 eV (Figure 1e), which is smaller than the ∼0.140 eV expected for CNT *K*-momentum excitons (*K*).^35^ This suggests that the emission peak may originate from CNT trions (*T*_CNT_). The PLE spectrum in Figure 1f indicates that the new emission peak is resonant with states near $X_{{\mathrm{WSe}}_{2}}$ , rather than with *E*_22_. In contrast, *E*_11_ is resonant with both $X_{{\mathrm{WSe}}_{2}}$ and *E*_22_.

The low-energy emission peak is observed consistently in multiple samples. Figure 1g compares four CNT/3L WSe_2_ heterostructures with (9,7), (10,5), (11,3), and (8,6) chiralities. Because the CNT bandgap depends on chirality, the *E*_11_ emission energy shifts from sample to sample. A similarly bright peak always appears below *E*_11_ in all four heterostructures, indicating that this trion-like emission is robust with respect to CNT chirality. Moreover, similarly bright peaks also emerge in (10,5) CNT heterostructures with WSe_2_ layers ranging from 1L to 4L (Figure S2), indicating no clear layer number dependence within this range.

A defining characteristic of CNT trions is the diameter *d* dependence of Δ*E*,^36,37^ reflecting the 1/*d* scaling (Figure 1h). The Δ*E* values observed here are close to those from surfactant-wrapped CNTs, suggesting a similar dielectric environment. Δ*E* is given by the equation:

1 $$\Delta E=\frac{A}{d}+\frac{B}{d^{2}}$$

where *A* and *B* are constants for the binding energy and the singlet–triplet splitting, respectively. *A* = 60 meV · nm and *B* = 67 meV · nm^2^ reproduce the measured trend, which is comparable with surfactant-wrapped CNTs;^37^ some deviations likely result from strain-induced shifts of *E*_11_ during the formation of these suspended structures.

As with the exciton transfer process, the appearance of the trion peak also strongly correlates with the band alignment. Every sample exhibiting a pronounced *T*_CNT_ peak displays type-I band alignment, whereas the type-II heterostructures show neither *E*_11_ nor *T*_CNT_ emission for excitation at the $X_{{\mathrm{WSe}}_{2}}$ energy (Figure S3).

### Trion Transfer in Mixed-Dimensional Heterostructures

Our experiments suggest that |*T*_CNT_⟩ is generated via a trion transfer process. Specifically, we propose that photoexcited trions in WSe_2_ lead to trion emission in CNTs, denoted by $\left|T_{{\mathrm{WSe}}_{2}}\right\rangle \to \left|T_{\mathrm{CNT}}\right\rangle$ . This process differs from free-carrier-induced trion emission where any excitation that populates |*E*_11_⟩ in a charged CNT can yield |*T*_CNT_⟩. Evidence supporting the absence of this free-carrier-based pathway includes the bright *E*_11_ emission (largely unquenched by free carriers; Figure 1c,d) and the lack of *T*_CNT_ under direct *E*_22_ excitation (Figure 1e,f). Instead, we propose that two transfer pathways coexist in the heterostructures: $\left|X_{{\mathrm{WSe}}_{2}}\right\rangle \to \left|E_{11}\right\rangle$ for excitons and $\left|T_{{\mathrm{WSe}}_{2}}\right\rangle \to \left|T_{\mathrm{CNT}}\right\rangle$ for trions. It is noted that $\left|X_{{\mathrm{WSe}}_{2}}\right\rangle$ and $\left|T_{{\mathrm{WSe}}_{2}}\right\rangle$ are nearly identical in energy (Figure 1f) at room temperature owing to the small trion binding energy (∼20 meV).^19^ However, the distinct spectral shapes (Figure 1f) suggest that two processes arise from different initial states.

In fact, spatially resolved PL excitation imaging can distinguish the exciton and trion transfer processes. We perform the excitation imaging measurements on the (9,7) CNT/3L WSe_2_ sample using a three-dimensional motorized stage (Figure 2a–c). The corresponding reflectivity image in Figure 2d maps the trench over the same area. No other abrupt morphological changes are resolved, confirming that the characterized area, including the CNT, is uniformly covered by the WSe_2_ flake. Under *E*_22_ excitation, the *E*_11_ emission profile matches the expected suspended CNT shape,^32,33^ whereas excitation at the $X_{{\mathrm{WSe}}_{2}}/T_{{\mathrm{WSe}}_{2}}$ energy produces a broadened PL excitation image. This enlarged image indicates that WSe_2_ A excitons excited at a distance funnel into the CNT after diffusion, acting as an exciton reservoir for exciting CNTs.^8^ Notably, under the same excitation energy, the *T*_CNT_ emission profile does not show such significant spatial broadening. Although $\left|T_{{\mathrm{WSe}}_{2}}\right\rangle$ and $\left|X_{{\mathrm{WSe}}_{2}}\right\rangle$ lie close in energy, the difference in the excitation images support a scenario in which $\left|T_{{\mathrm{WSe}}_{2}}\right\rangle$ is initially excited in the trion transfer process and functions as a trion reservoir.

**Figure 2:**
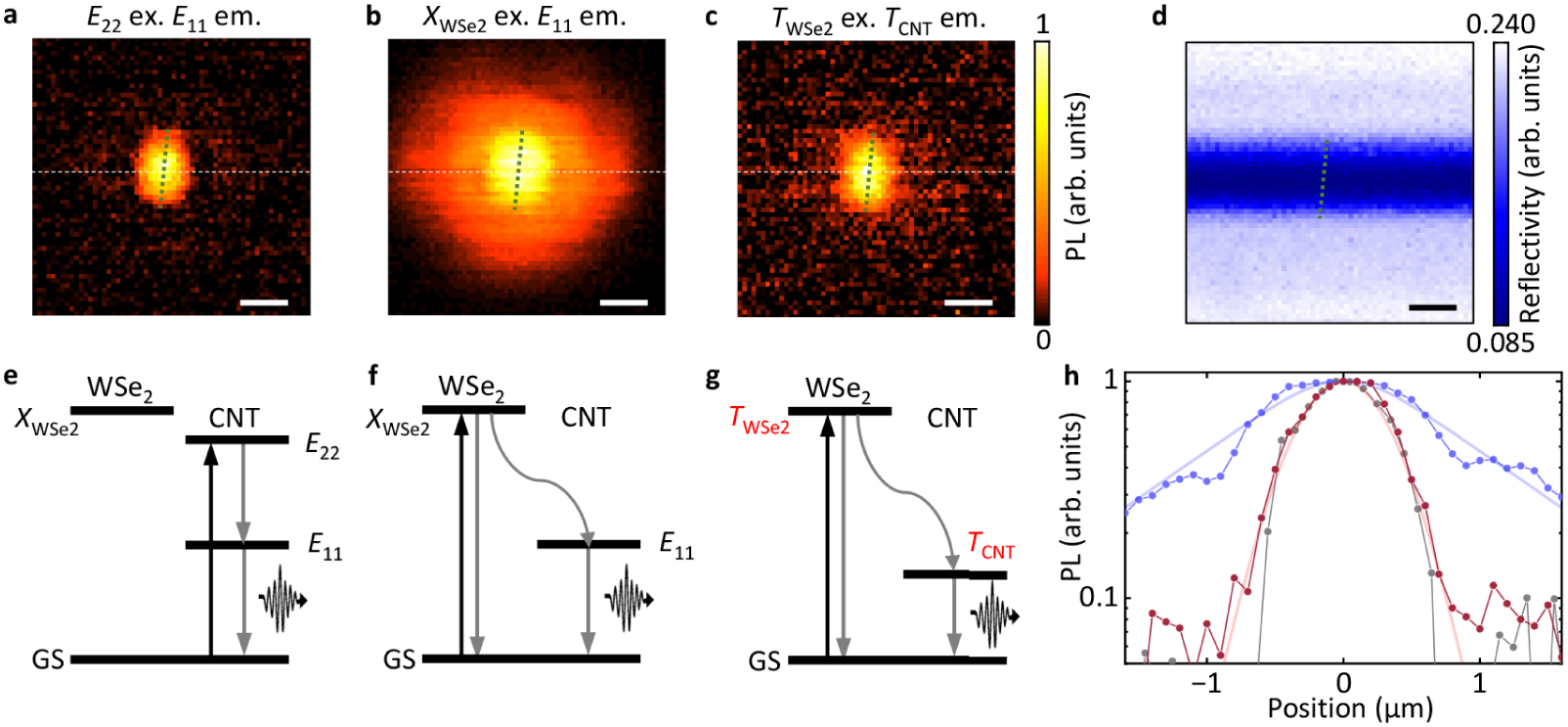
(a–c) Normalized PL intensity maps from the (9,7) CNT/3L WSe_2_ sample whose PL spectrum is shown in Figure 1g. The PL images are constructed by integrating PL emission over a 30 meV-wide spectral window centered at *E*_11_ energy (a, b) and *T*_CNT_ energy (c). The excitation is at *E*_22_ (1.459 eV, a), $X_{{\mathrm{WSe}}_{2}}$ (1.653 eV, b), and $T_{{\mathrm{WSe}}_{2}}$ (1.653 eV, c). The excitation power is 10 μW. (d) The corresponding reflectivity image. The excitation is at *E*_22_ (1.459 eV). (e–g) Energy level diagrams showing the three processes occurring in (a–c), respectively. GS indicates the ground state. (h) Line profiles taken from (a–c), as indicated by the white broken lines. The gray, blue, and red symbol-line plots are the experimental results from (a), (b), and (c). The blue and red lines are corresponding fits. The scale bars in (a–d) represent 1 μm. The CNT is indicated by the broken green lines in (a–d).

This difference in spatial broadening highlights distinct diffusion processes for $\left|T_{{\mathrm{WSe}}_{2}}\right\rangle \to \left|T_{\mathrm{CNT}}\right\rangle$ and $\left|X_{{\mathrm{WSe}}_{2}}\right\rangle \to \left|E_{11}\right\rangle$ (Figure 2e–g). To quantify the diffusion lengths, we fit the PL line profiles in Figure 2h with numerical solutions to the steady-state, one-dimensional diffusion equation:

2 $$D\frac{d^{2}n\left(x\right)}{dx^{2}}-\frac{n\left(x\right)}{\tau }+\frac{G}{\sqrt{2\pi r^{2}}}\exp\left(-\frac{2x^{2}}{r^{2}}\right)=0$$

where *n* is the trion (exciton) density that directly reflects the PL intensity, *x* is the position along the trench, *D* is the diffusion coefficient, τ is the lifetime, and *r* = 0.58 μm is the radius of the Gaussian laser profile. The best-fit simulation yields a diffusion length $L=\sqrt{D\tau }=1.0\mu m$ for A excitons and only 0.15 μm for trions, in agreement with the slight trion spatial broadening beyond the laser spot. The results indicate that the trion diffusion length is approximately 1 order of magnitude smaller than that of the A exciton, which is consistent with previous studies of TMDCs reporting exciton and trion diffusion lengths of 1.5 and 0.3 μm, respectively.^38^

The relatively short trion diffusion length in WSe_2_ limits $\left|T_{{\mathrm{WSe}}_{2}}\right\rangle$ to a distance of ∼150 nm around the CNT that may further contribute to the emission at |*T*_CNT_⟩ through transfer. It is, however, still significantly more efficient than the direct excitation process, which is strictly confined to the ∼1 nm CNT diameter. Moreover, we observe a pronounced trion reservoir effect in the time domain, as revealed by time-resolved PL (Figure S4). In a gate-doped suspended CNT under *E*_22_ excitation, |*T*_CNT_⟩ decays with a 26 ps lifetime. In contrast, in the heterostructure under $T_{{\mathrm{WSe}}_{2}}$ excitation energy, it increases to 281 ps. Specifically, $\left|T_{{\mathrm{WSe}}_{2}}\right\rangle$ with a lifetime of 521 ps continuously funnels into the CNT, acting as a trion reservoir in the time domain and extending the overall decay. Although transferred trions *T*_CNT_ in the heterostructure exhibits a shorter lifetime than $T_{{\mathrm{WSe}}_{2}}$ in pristine WSe_2_, this does not indicate the emergence of nonradiative pathways. In fact, because CNTs are intrinsically undoped and defect-free, their bright excitons possess near-unity radiative quantum efficiency.^39^ Trions transferred to the CNT bypass nonradiative loss channels in WSe_2_ and could achieve similarly high quantum efficiency. By performing Monte Carlo simulations, we estimate that ∼20% of trions excited in WSe_2_ transfer to the CNT (Figure S5), which is a significant fraction given the dimensional mismatch here. Moreover, trion transfer time is estimated to be 1.3 ps, which is comparable with the fastest exciton transfer processes.^8,9,40^

Trion transfer shares several features with exciton transfer, including comparable excitation energies and prolonged decay curves. Both processes exhibit linear excitation polarization independence (Figure S6), reflecting the two-dimensional nature of WSe_2_ excitonic states. However, intercrossing processes between trions and excitons should not be allowed. Under the charge-conservation rule, transitions such as $\left|T_{{\mathrm{WSe}}_{2}}\right\rangle \to \left|E_{11}\right\rangle$ and $\left|X_{{\mathrm{WSe}}_{2}}\right\rangle \to \left|T_{\mathrm{CNT}}\right\rangle$ are not permitted. In addition, since bright excitons are spin-singlets and trions are spin-doublets, spin conservation also restricts transitions between quasi-particles of disparate spins. Consequently, no clear signature of population intermixing between excitons and trions is observed.

### Trion Transfer Overcoming Free-Charge-Induced Nonradiative Limits

Unlike conventional trion formation which relies on free carriers in the emitter, trion transfer exploits the reservoir effect that promises brighter trion emission. To examine the increased emission efficiency, we measure the excitation power dependence in a (10,5) CNT/3L WSe_2_ sample (Figure 3a). At a low power of 2 μW, *T*_CNT_ is much weaker than *E*_11_. With increasing power, *T*_CNT_ increases more substantially than *E*_11_. As shown in Figure 3b, *E*_11_ saturates at high powers due to exciton–exciton annihilation (EEA), whereas *T*_CNT_ increases linearly over the entire range. The results indicate that trion emission via transfer largely avoids the nonlinear inefficiency and suggest that trion–trion annihilation is minimal, possibly owing to electrostatic repulsion among trions.

**Figure 3:**
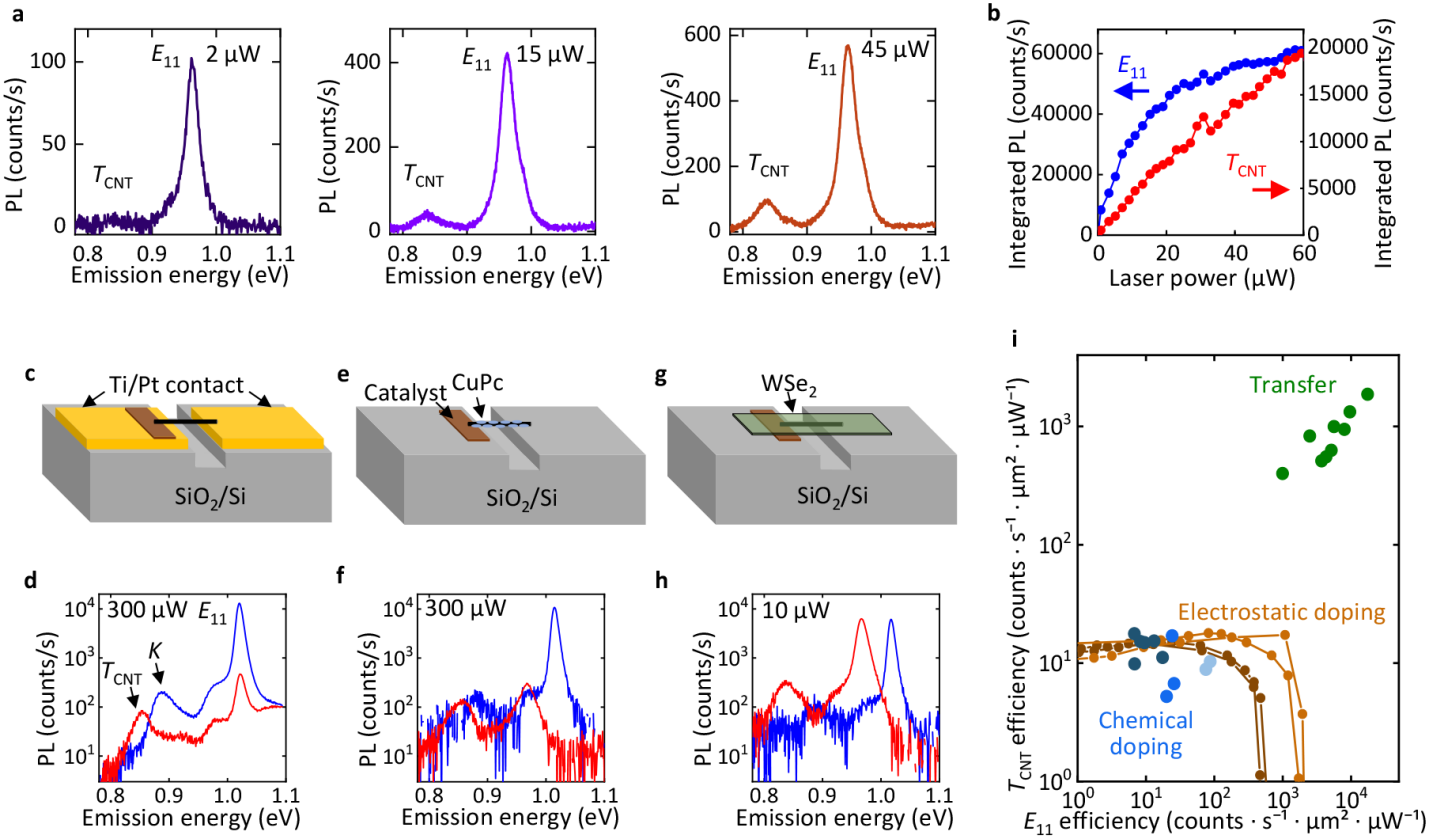
(a) PL spectra of the (10,5) CNT/3L WSe_2_ sample at powers of 2, 15, and 45 μW from left to right. The excitation is at $X_{{\mathrm{WSe}}_{2}}/T_{{\mathrm{WSe}}_{2}}$ energy. (b) Integrated PL intensity as a function of the laser power for *E*_11_ and *T*_CNT_, respectively. The PL emission is integrated over a 50 meV-wide spectral window centered at the *E*_11_ or *T*_CNT_ energies. (c) Schematic image of the suspended gated-CNT structure. (d) PL spectra of the (10,5) gated CNT without (blue) and with *V*_g_ = −0.8 V (red). The excitation power is 300 μW and the excitation energy is *E*_22_. (e) Schematic image of the CNT/CuPc hybrid. (f) PL spectra of the pristine (10,5) CNT (blue) and the (10,5) CNT/26 nm-thick CuPc hybrid (red). The excitation powers are 100 μW for the pristine CNT, 300 μW for the CNT/CuPc hybrid, and the excitation energy is *E*_22_. (g) Schematic image of the CNT/WSe_2_ heterostructure. (h) PL spectra of the (10,5) CNT before (blue) and after the formation of the heterostructure with a 1L WSe_2_ flake (red). The excitation power is 10 μW and the excitation energy is *E*_22_ for the pristine tube and $X_{{\mathrm{WSe}}_{2}}/T_{{\mathrm{WSe}}_{2}}$ for the heterostructure. (i) *T*_CNT_ efficiency and *E*_11_ efficiency from the three different structures. Green dots are from nine different CNT/WSe_2_ heterostructures with an excitation power of 10 μW at $X_{{\mathrm{WSe}}_{2}}/T_{{\mathrm{WSe}}_{2}}$ energy. Brown symbol-line plots are from four suspended gated-CNT samples obtained by sweeping *V*_g_ from 0 V to the positive side (light-brown two measured at 100 μW and dark-brown two at 300 μW). The excitation energy is *E*_22_. Blue dots are from different CNT/CuPc samples. Light, medium, and dark blue indicate CuPc deposition thicknesses of 7, 16, and 26 nm, respectively. The excitation power is 300 μW and the excitation energy is *E*_22_.

To highlight the efficiency of transfer-based trion emission, we compare it with two standard trion-generation approaches: electrostatic doping and chemical doping (Figure 3c–h). In the electrostatic method,^41^ we use a suspended (10,5) CNT field-effect transistor and apply a back-gate voltage *V*_g_ (Figure 3c). Under *E*_22_ excitation and zero gate bias, the PL spectrum exhibits a bright *E*_11_ peak at 1.020 eV and a side peak *K* at 0.887 eV. At *V*_g_ = −0.8 V, free carriers introduced into the CNT quench both *E*_11_ and *K*, while *T*_CNT_ emerges at 0.856 eV (Figure 3d). Because free-carrier-driven Auger recombination depletes excitons, the resulting trion emission remains weak and requires a high laser power of 300 μW to clearly resolve.

A similar phenomenon occurs with chemical doping. Depositing copper phthalocyanine (CuPc) onto suspended CNTs forms CNT/CuPc hybrids,^36^ introducing free carriers (Figure 3e). Compared to a pristine (10,5) CNT, the (10,5) CNT/CuPc hybrid shows a strongly quenched *E*_11_ and an emergence of *T*_CNT_ (Figure 3f). Increasing the CuPc thickness from 7 to 26 nm systematically raises the doping level, thereby enhancing trion emission at the expense of *E*_11_ emission (Figure 3i). As with electrostatic doping, trion emission remains modest under the high power of 300 μW.

In contrast, transfer-induced trions exhibit a significantly stronger emission. Figure 3h shows PL spectra from a (10,5) CNT before and after transferring a monolayer WSe_2_ flake (Figure 3g). Under direct *E*_22_ excitation, the pristine CNT exhibits a clear *E*_11_ peak. After forming the heterostructure and exciting at the $X_{{\mathrm{WSe}}_{2}}/T_{{\mathrm{WSe}}_{2}}$ energy, we observe bright *E*_11_ and *T*_CNT_ peaks. The absence of free carriers in the CNT, combined with the reservoir effect for excitons and trions, yields the preserved *E*_11_ emission and the significantly increased *T*_CNT_ emission at a low laser power of 10 μW.

To quantitatively compare these mechanisms, we define emission efficiencies for *T*_CNT_ and *E*_11_ by normalizing each integrated PL intensity to the excitation power density (Figure 3i) (see Methods and the Supporting Information for details of the collection efficiency normalization). In four electrostatically doped CNTs, sweeping *V*_g_ initially increases *T*_CNT_ efficiency, while *E*_11_ efficiency is inevitably quenched. By further increasing *V*_g_, *T*_CNT_ efficiency eventually saturates and then declines. Such saturation behavior with respect to carrier density is widely observed in trion-emitting systems,^42–44^ suggesting an intrinsic efficiency limit. Notably, at zero *V*_g_ condition, *E*_11_ efficiency is lower than in CNT/WSe_2_ heterostructures. This difference arises from efficient exciton transfer in the heterostructures and strong EEA under high laser power in gated CNTs; the latter can be largely avoided by reducing the excitation from 300 μW to 100 μW. Meanwhile, at high *V*_g_ condition where *E*_11_ efficiency is severely degraded, *T*_CNT_ efficiency is insensitive to laser power, consistent with the absence of trion–trion annihilation. CNT/CuPc samples follow a similar trend with carrier density, which is modulated by CuPc thickness. We also note that the results are more scattered due to strain and morphological variations.

Transfer-based trion emission in all CNT/WSe_2_ samples allows *E*_11_ and *T*_CNT_ to coexist and scale together. Remarkably, the best samples achieve trion emission efficiencies more than 2 orders of magnitude above the limits of doping-based methods. Given that exciton transfer proceeds via a direct tunneling mechanism, it is plausible that trion transfer similarly involves simultaneous tunneling of an additional charge (Figure S3), and an efficient tunneling pathway enhances both processes. After trion emission, the extra charge likely tunnels back to WSe_2_ under equilibrium conditions and the CNT remains undoped, as supported by the lack of any photodoping signature. Such charge tunneling is expected to be very fast since there is no clear sign of saturation in the trion transfer process even at high excitation power (Figure 3b). It implies that the charge tunneling back to WSe_2_ is faster than the trion transfer process with a time scale of 1.3 ps and occurs on a subpicosecond time scale. Moreover, because the reservoir effect for both excitons and trions depends strongly on diffusion, WSe_2_ flakes with fewer scattering centers should enhance both emissions, explaining the observed correlation in the generation of |*E*_11_⟩ and |*T*_CNT_⟩. Improvements of tunneling efficiency and WSe_2_ uniformity are thus expected to further increase trion emission, marking a conceptual difference from conventional doping-based approaches.

### Trion Transfer in Free-Charge-Modulated Mixed-Dimensional Heterostructures

Overcoming free-charge-induced limits suggests that free charges do not play a distinct role in the trion transfer process. To gain a comprehensive understanding of this mechanism, we deliberately introduce free carriers into the mixed-dimensional heterostructures via either field-effect gating or substitutional doping. We first achieve controlled doping in the nanotubes by fabricating a suspended (10,5) CNT field-effect transistor and transferring a 3L WSe_2_ flake onto it (Figure 4a,b). As natural WSe_2_ crystals contain defects with a density of 10^12^ cm^–2^,^45^ defect-induced gap states weaken the gate bias modulation effect at the suspended WSe_2_ Fermi level. In contrast, the CNTs are characterized to be free of defects so that gate bias would effectively modulate the suspended CNT Fermi level.^41^

**Figure 4:**
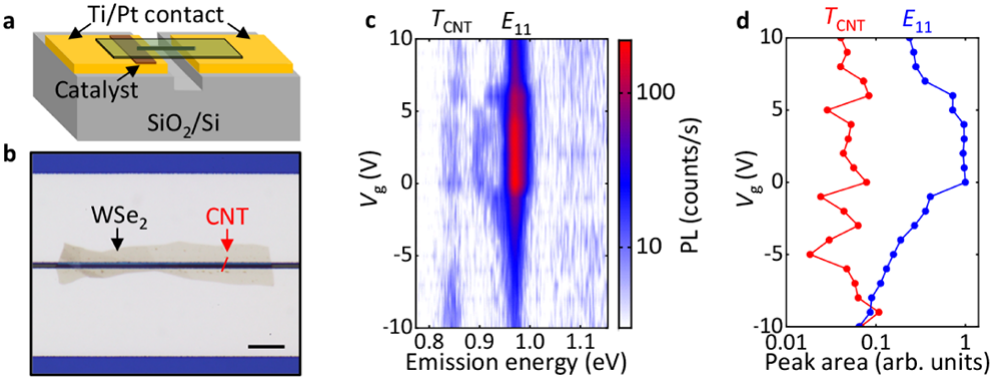
(a) A schematic of a suspended gated CNT/WSe_2_ heterostructure. (b) An optical image of the (10,5) CNT/3L WSe_2_ device. The scale bar represents 10 μm. (c) PL spectra as a function of gate voltage. The excitation power is 2 μW and the excitation energy is $X_{{\mathrm{WSe}}_{2}}/T_{{\mathrm{WSe}}_{2}}$ at 1.642 eV. (d) PL peak area for *T*_CNT_ (red) and *E*_11_ (blue) in (c) as a function of gate voltage. The peak area is obtained by Lorentzian fitting at each *V*_g_.

Figure 4c shows the gate-dependent PL spectra of this heterostructure under $X_{{\mathrm{WSe}}_{2}}/T_{{\mathrm{WSe}}_{2}}$ excitation. *E*_11_ is strongly quenched at high gate bias, indicating an effective field-effect modulation of the nanotube. We note that *E*_11_ in the transistor configuration is slightly quenched at a gate bias of 0 V compared with the pristine CNT/WSe_2_ heterostructures, likely due to additional charging of the CNT through the metal in contact with the WSe_2_. Consequently, the charge-neutral point shifts from 0 V to *V*_g_ = 2.9 V, indicating p-type doping by defects in WSe_2_. Remarkably, *T*_CNT_ emerges at the charge-neutral point, again underscoring the key feature of transfer-induced trions: they do not require free carriers in the CNT.

Such a “pure” trion flux free of extra carriers is highly relevant for trion-based device applications, which rely on manipulating spin and charge of trion qubits. Unlike free carriers, the excitons cogenerated do not share the same spin or charge as trions, and can be further excluded by exploiting selection rules in the transfer process. By either lowering the operating temperature of the heterostructure transistor or choosing a donor material where exciton and trion energies are more distinctly separated, selective trion excitation can be achieved that leads to an even purer trion flux.

We also observe that trion emission remains largely insensitive to *V*_g_ over the entire range, as clearly indicated by the *T*_CNT_ peak area versus *V*_g_ in Figure 4d. In another sample, *E*_11_ is quenched by ∼94% at large negative *V*_g_, yet *T*_CNT_ remains unquenched (Figure S7). Such a charge-density robustness distinguishes trion transfer not only from charge-induced trion emission but also from most other optical processes, where high carrier densities typically quench PL.

After examining the free-carrier effect on the CNT acceptor in the trion transfer process, we next focus on the WSe_2_ donor by introducing Nb substitutional doping (Figure 5a; see Methods). Incorporating Nb at ∼8.8 × 10^18^ cm^–3^ produces a nondegenerate p-doped WSe_2_ flake.^46^ When the 1L flake is transferred onto a (11,3) CNT, the *E*_11_ peak redshifts by 0.045 eV, and the emission is quenched by more than 90% (Figure 5b). The doping in WSe_2_ shifts its Fermi level, and charges move across the interface to align the Fermi levels when forming the heterostructure. Such ground-state charge transfer dopes the CNT and thereby quenches its PL.

**Figure 5:**
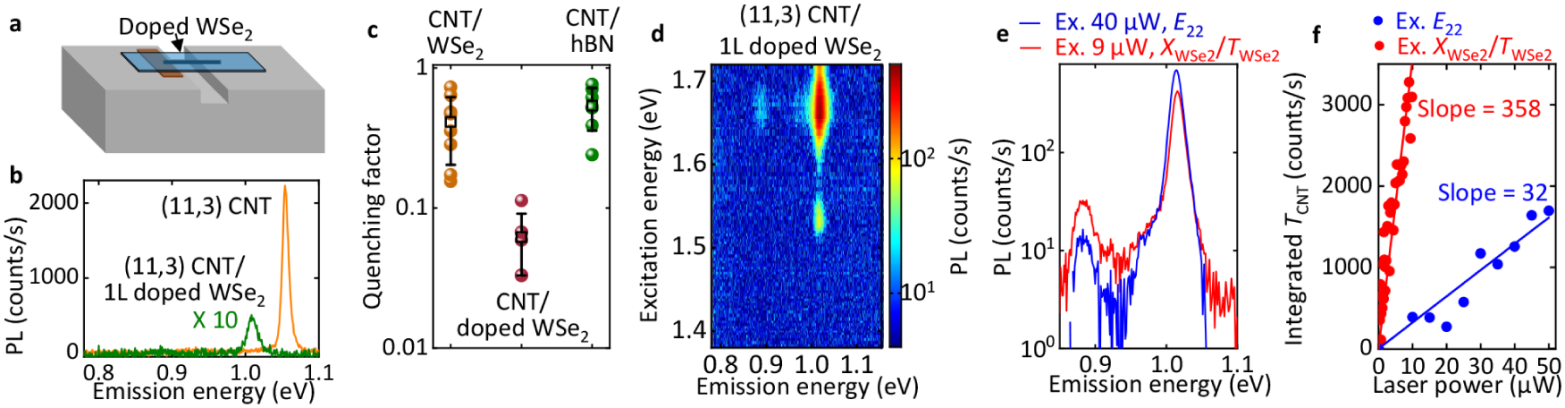
(a) A schematic of a suspended CNT/WSe_2_ heterostructure. (b) PL spectra of the (11,3) CNT before (orange) and after the formation of the heterostructure with a 1L doped WSe_2_ flake (green). The excitation power is 10 μW and the excitation energy is *E*_22_. (c) Quenching factor *Q* for three structures. Error bars are the standard error of the mean. (d) The PLE map of the (11,3)/1L doped WSe_2_ heterostructure. The excitation power is 10 μW. (e) The PL spectra of the (11,3)/1L doped WSe_2_ heterostructure at *E*_22_ (1.540 eV, 40 μW, blue) and $X_{{\mathrm{WSe}}_{2}}/T_{{\mathrm{WSe}}_{2}}$ (1.634 eV, 9 μW, red), respectively. (f) Integrated PL intensity of *T*_CNT_ as a function of the laser power at *E*_22_ (blue) and $X_{{\mathrm{WSe}}_{2}}/T_{{\mathrm{WSe}}_{2}}$ (red) from the (11,3)/1L doped WSe_2_ heterostructure. The *T*_CNT_ emission is integrated over a 50 meV-wide spectral window centered at the peak energy.

To quantify this quenching, we define a quenching factor *Q* as the *E*_11_ PL intensity under *E*_22_ excitation with respect to the same CNT prior to heterostructure formation. Figure 5c compares *Q* for three heterostructures: CNT/WSe_2_, CNT/doped WSe_2_, and CNT/hexagonal boron nitride (hBN). Because bulk hBN serves as an excellent insulator for CNTs,^47^ the quenching in CNT/hBN mainly reflects other factors such as strain from the transfer process rather than charge transfer. In CNT/WSe_2_, many samples show similar high *Q* values as in CNT/hBN, again confirming negligible free charges in the CNT. A few samples exhibit lower *Q*, likely due to significant strain from flexible thin WSe_2_ flakes. In contrast, CNT/doped WSe_2_ samples yield an average *Q* of only 0.062, confirming strong quenching by free carriers through the charge transfer process.

Despite this charge-induced quenching, both exciton and trion transfer persist in CNT/doped WSe_2_ heterostructures. As indicated in the PLE map in Figure 5d, *E*_11_ emission at 1.018 eV and *T*_CNT_ emission at 0.883 eV are resonantly excited at the $X_{{\mathrm{WSe}}_{2}}/T_{{\mathrm{WSe}}_{2}}$ energy of 1.670 eV. Since the CNT is now charged, we also expect trion emission under direct *E*_22_ excitation. Indeed, a trion peak appears under *E*_22_ excitation energies (Figure 5e), confirming charge transfer. However, even at a higher laser power of 40 μW, *T*_CNT_ emission via *E*_22_ remains much weaker than via trion transfer at 9 μW, consistent with the higher efficiency of the latter (Figure 5f). From the power dependence in Figure 5f, the trion peak increases linearly with laser power, showing a slope of 358 counts · s^–1^ · μW^–1^ under $X_{{\mathrm{WSe}}_{2}}/T_{{\mathrm{WSe}}_{2}}$ excitation, consistent with the behavior in CNT/WSe_2_ samples (Figure 3a). In contrast, direct *E*_22_ excitation yields a slope of only 32 counts · s^–1^ · μW^–1^, over an order of magnitude smaller.

Although transfer-induced trion emission in the CNT/doped WSe_2_ sample far exceeds that of free-carrier-based trion generation methods, it is weaker than in the undoped case (Figure 3h and Figure 5e). Note that doping the CNT itself does not necessarily degrade trion transfer (Figure 4). Instead, we attribute the reduced trion emission to the impact of Nb dopants on the spatial reservoir effect for trions in WSe_2_. PL excitation images (Figure S8) show much reduced diffusion, with the exciton diffusion length decreasing to 0.12 μm and the trion length unresolvable. Because trions carry net charge and have relatively small binding energies, they are expected to be more susceptible to scattering, likely restricting their diffusion to 10–100 nm. Consequently, the diminished reservoir effect can attenuate trion emission compared to undoped WSe_2_.

The coexistence of charge, exciton, and trion transfer in the doped 1D/2D heterostructures offers broader insights for other material systems. We anticipate that a variety of low-loss 1D or 0D emitters, such as nanotubes, nanowires, or quantum dots, could likewise accept trions from higher-dimensional donors efficiently without suffering strong nonradiative recombinations. It also suggests that in heterostructures such as 2D/2D assemblies where no dimensional heterogeneity exists, trion reservoir effect may be weaker. However, the key hallmarks of trion transfer, resonance with the excitation states in the donor and insensitivity to the free carriers in the acceptor, should still hold, which provides a versatile route to trion generation and manipulation.

A reservoir-fed high-density trion population confined to a 1D channel raises the prospect of superfluorescence, where the cooperative emission scales as the square of the participating population. Trion-mediated optical gain can couple readily to small-mode-volume, high-Q cavities for cavity quantum electrodynamical effects and low-threshold lasing. With a 1D trion flux, strong trion–trion repulsion is anticipated, which maybe manifested in the negligible trion–trion annihilation in Figure 3b. As charged composite Fermions, trions experience long-range Coulomb repulsion and an effective Fermi pressure that exceed the short-range exchange and phase-space-filling interactions governing neutral excitons. Under these Fermi-pressure-driven constraints, a trion flux may support long-range coherent transport and enable optoelectronic functionalities with both electrical and optical readout. Moreover, their internal spin configurations could serve as qubit degrees of freedom in quantum computing, with further effort on robust initialization, coherent control, and high-fidelity readout.

## Conclusions

In conclusion, we have demonstrated a conceptually new optical mechanism, trion transfer, in mixed-dimensional heterostructures formed by 1D CNTs and 2D WSe_2_. By circumventing carrier doping in the CNT, this process overcomes the typical nonradiative Auger limitations and yields trion emission efficiencies exceeding conventional methods by more than 2 orders of magnitude. Detailed measurements, including photoluminescence excitation, spatial mapping, and time-resolved PL, reveal how trions photoexcited in WSe_2_ can diffuse and transfer into the CNT without introducing free carriers. This mechanism not only preserves bright exciton emission but also remains robust under CNT gating or WSe_2_ doping. Our findings establish that dimensional heterogeneity—a 1D acceptor combined with a 2D donor—provides a unique trion reservoir effect that can be harnessed for efficient trion generation. Extending these concepts to other low-dimensional systems could open new opportunities in many-body excitonic physics, spin/valleytronics, and advanced optoelectronics, paving the way for novel trion-based devices.

## Methods

### Air-Suspended Carbon Nanotube Growth

To prepare air-suspended CNTs, we start from silicon dioxide (SiO_2_)/silicon (Si) substrates with prefabricated trenches.^32,33^ Electron-beam lithography (EBL) is used to pattern alignment markers and trenches on the Si substrate, followed by dry etching to form trenches approximately 900 μm in length and 0.5–2.0 μm in width. A thermal oxidation step then grows a 60–70 nm SiO_2_ film inside the trenches. Next, another round of EBL defines the areas where an iron (Fe) film (∼1.5 Å thick) is deposited by electron-beam evaporation as a catalyst for CNT growth. Finally, CNTs are synthesized by alcohol chemical vapor deposition at 800 °C for 1 min. By optimizing the Fe thickness, we predominantly obtain isolated and high-quality CNTs. For heterostructure fabrication with WSe_2_, we select isolated, defect-free, chirality-identified CNTs with lengths of 1.0–2.0 μm. Specifically, the tubes are confirmed to exhibit bright PL emission with a small line width of ∼10 meV, a smooth suspended single-tube shape in PL imaging without any trapping or quenching sites, and a high linear polarization degree over 90%.

### Anthracene Crystal Growth

Anthracene crystals used for stamping WSe_2_ flakes onto CNTs are grown by in-air sublimation.^7,8^ Anthracene powder is placed on a glass slide maintained at 80 °C. A second glass slide, typically ∼1 mm above the anthracene source, collects the sublimated material. Thin, large-area single crystals grow on the upper slide over 10 h. To promote the formation of thin, large-area crystals, ink patterns from a commercial marker are applied to the glass, inhibiting 3D crystal nucleation.

### 2D Materials Preparation

Natural WSe_2_ crystals are purchased from HQ Graphene, and their quality is characterized by electrical measurement.^48^ They exhibit a relatively high defect density of ∼10^12^ cm^–2^ but remain nearly neutral with only slight carrier doping because most defects are deep-level states. Nb-doped WSe_2_ crystals are synthesized through chemical vapor transport.^46^ In this growth, powders of W, Nb, and Se are uniformly distributed within a quartz ampule and placed into a tube furnace. Nb-doped WSe_2_ single crystals grow across the ampule.

### WSe_2_ Transfer Using Anthracene Crystals

WSe_2_ flakes are mechanically exfoliated onto standard 90 nm SiO_2_/Si substrates. Their layer thickness is determined by optical contrast. A polydimethylsiloxane (PDMS) stamp supported on a glass slide is used to pick up a single anthracene crystal, forming an anthracene/PDMS stamp. The anthracene crystal then collects the targeted WSe_2_ flake from the substrate by pressing the stamp onto the flake and withdrawing it rapidly (>10 mm/s). Next, the same stamp is aligned over a chirality-identified CNT, whose location is determined via previous measurements, and peeled off slowly (<0.2 μm/s) so the anthracene/WSe_2_ stack adheres to the receiving substrate. The anthracene crystal is then removed by sublimation in air at 110 °C for ∼10 min, leaving behind a clean, fully suspended CNT/WSe_2_ heterostructure. This dry transfer protocol minimizes contamination, and the anthracene crystal mechanically protects both the CNT and the WSe_2_ flake during transfer.^7,8^

### CNT/hBN Heterostructure Preparation

The thick hBN flakes ranging from 20 to 90 nm are first prepared on PDMS by mechanical exfoliation from crystals supplied by NIMS, and then transferred onto the target CNTs at 120 °C using a micromanipulator system.^47^

### CuPc Deposition on CNTs

The chip with CNTs is placed in a vacuum chamber for the evaporation of CuPc (Sigma-Aldrich) and is maintained at about 80 °C for 10 min to remove adsorbed molecules on the CNTs.^36^ CuPc molecules are deposited on suspended CNTs in the chamber using an evaporator heated to 480–520 °C under a vacuum of less than 10^–4^ Pa. A glass slide is also placed in the chamber to quantify the deposition thickness from the absorbance of the CuPc peak. Calibration is performed by measuring the actual thickness with a surface profiler for two CuPc films with thicknesses of 80 and 167 nm. Three samples with different deposition amounts are prepared by changing the evaporation time, yielding nominal thicknesses on the substrate of 7, 16, and 26 nm.

### Suspended CNT Field-Effect Transistor Fabrication

The structures are fabricated on p-type Si substrates (resistivity ∼15 ± 5 mΩ · cm) coated with a 100 nm oxide layer.^41^ Trenches (500 nm deep, 0.4–1.6 μm wide) are formed by EBL and dry etching. The substrates are then oxidized at 900 °C for 1 h, creating an additional 20 nm of SiO_2_ in the trenches. A second EBL step defines electrode patterns, followed by electron-beam evaporation of Ti (2 nm)/Pt (20 nm). After liftoff, a third EBL step patterns catalyst regions near the trenches, and CNTs are subsequently grown to span the trenches. The resulting suspended field-effect transistors enable efficient carrier-density modulation in the suspended CNT region, as confirmed by electrical characteristics and PL spectroscopy.^41,49^

### Photoluminescence Measurements

All PL measurements are performed at room temperature in a custom-built confocal microscopy setup purged with dry nitrogen gas. A Ti:sapphire laser, tunable in wavelength and operated in continuous-wave mode, serves as the excitation source. The laser power is controlled by neutral-density filters, and the beam is focused onto the sample via an objective lens (numerical aperture NA = 0.65, working distance = 4.5 mm), producing a 1/e^2^ beam diameter of ∼1.16 μm. A confocal pinhole sets the collection spot size to ∼5.4 μm in diameter. Emitted PL is collected by the same objective, dispersed by a spectrometer with a 150 lines/mm grating (dispersion ∼0.52 nm/pixel at 1340 nm), and detected with a liquid-nitrogen-cooled InGaAs diode array (1024 pixels). The reflected beam from the sample traces back the same path and is detected by a silicon photodiode. The excitation polarization angle is always adjusted to be parallel to the CNT axis unless specifically mentioned. For spatially resolved imaging in Figure 2, a three-dimensional motorized stage scans the heterostructure to generate PL excitation images. Because the collection spot exceeds the excitation spot, photons emitted outside the immediate laser focus are also collected, and the laser spot size ultimately sets the spatial resolution. Consequently, these images reflect the excitation efficiency profiles for the PL and help identify nonlocal excitation phenomena. For the CNT/CuPc samples in Figure 3 and gated CNT/WSe_2_ samples in Figure 4, we use a similar setup with an objective lens of NA = 0.8 (working distance = 3.4 mm), producing a 1/e^2^ beam diameter of ∼0.94 μm. Since numerical aperture for the PL measurement system of CNT/CuPc samples is different from the other two structures in Figure 3i, emission collection efficiency is also considered in *T*_CNT_ efficiency and *E*_11_ efficiency evaluation (see the Supporting Information for details of the collection efficiency normalization).

## Supplementary Material



## Notes

Additional data and figures: PL spectra from suspended WSe_2_ flakes; PL spectra from heterostructures with varying WSe_2_ thickness; PLE maps from different heterostructures; time-resolved PL measurements to estimate trion and exciton transfer ratios; Monte Carlo simulation of the transfer process; excitation polarization measurements on the heterostructure; collection efficiency normalization in TCNT efficiency comparison; trion transfer in another gated CNT/WSe_2_ heterostructure; reduced spatial reservoir effect in CNT/Nb-doped WSe_2_ heterostructures (PDF)
