# Supplementary material for: Trion Transfer in Mixed-Dimensional Heterostructures
Source: ACS Nano. 2026 Apr 2;20(14):10933–42. doi: 10.1021/acsnano.5c17799 (PMC13085913; doi:10.1021/acsnano.5c17799)
Supplement: Supplementary file 1 [file nn5c17799_si_001.pdf]

Supporting Information for

Trion transfer in mixed-dimensional heterostructures

Nan Fang[1,2], Ufuk Erkılıç[1,2], Yih-Ren Chang[1,2], Shun Fujii[2,3], Daiki Yamashita[2,4], Chee Fai Fong[1,2], Satoru Morito[5], Kaito Kanahashi[6], Takashi Taniguchi[7], Kenji Watanabe[8], Keiji Ueno[5], Kosuke Nagashio[6], Yuichiro K. Kato[1,2]

[1]Nanoscale Quantum Photonics Laboratory, RIKEN Pioneering Research Institute, Saitama 351-0198, Japan

[2]Quantum Optoelectronics Research Team, RIKEN Center for Advanced Photonics, Saitama 351-0198, Japan

[3]Department of Physics, Faculty of Science and Technology, Keio University, Yokohama, 223-8522, Japan

[4]Photonics-Electronics Integration Research Center, National Institute of Advanced Industrial Science and Technology (AIST), Ibaraki, 305-8568, Japan

[5]Department of Chemistry, Saitama University, Saitama 338-8570, Japan

[6]Department of Materials Engineering, The University of Tokyo, Tokyo 113-8656, Japan

[7]Research Center for Materials Nanoarchitectonics (MANA), National Institute for Materials Science, Ibaraki 305-0044, Japan

[8]Research Center for Electronic and Optical Materials, National Institute for Materials Science, Ibaraki 305-0044, Japan

Supplementary Note 1:

Layer number dependence of PL from suspended WSe_2 flakes

The PL spectra of suspended 1L–4L WSe_2 flakes are shown in Fig. S1. For the 1L WSe_2 flake, a clear trion peak (1.633 eV) appears on the low-energy side of the A exciton (1.659 eV), demonstrating that our natural WSe_2 crystals are slightly and unintentionally doped. The trion binding energy is 26 meV, which is consistent with previous studies. In 2–4L WSe_2 flakes, the low-energy side of PL spectra is dominated by momentum-indirect excitons, which overlap with the energy range where trions would appear. This makes a reliable deconvolution of trion contributions in 2–4L WSe_2 difficult.

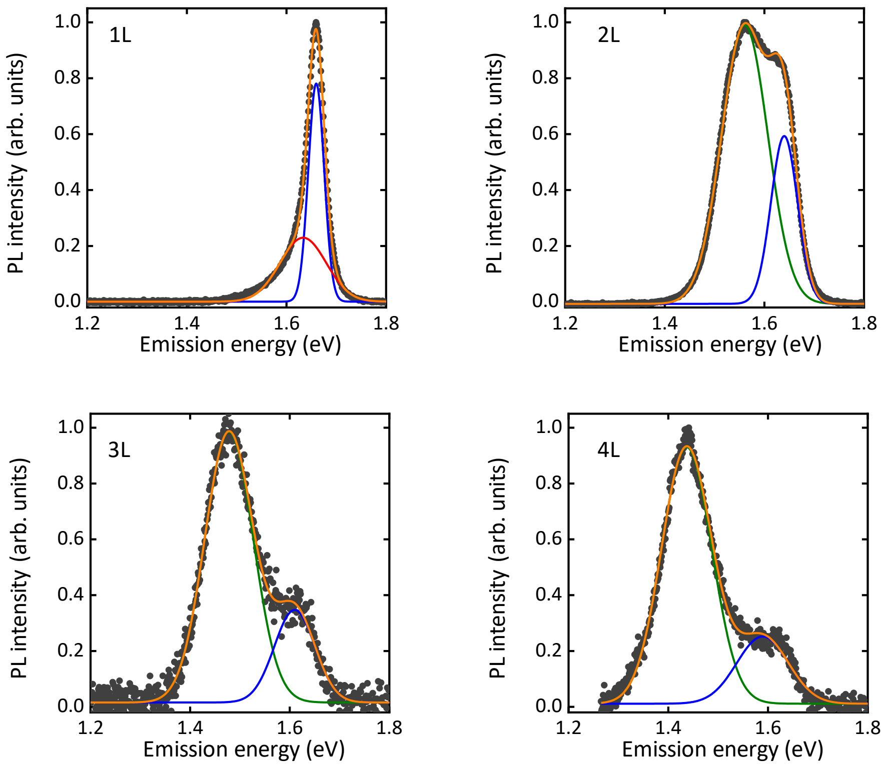

Fig. S1 | PL spectra from the suspended WSe_2 flakes with different layer numbers. The excitation power is 10 μW and the excitation wavelength is 532 nm. Black dots are experimental data. Solid lines indicate Gaussian fits decomposed into contributions from the WSe_2 A exciton (blue), trion (red, monolayer only), and momentum-indirect excitons (green), and the orange line indicates the total fitted PL spectrum.

Supplementary Note 2:

WSe_2 layer number dependence on trion transfer process

We observe consistent trion emission peaks from transfer process in heterostructures with vairous WSe_2 layer number. Fig. S2 shows PL spectra for (10,5) CNT/WSe_2 heterostructures with WSe_2 layers ranging from 1L to 4L. In all samples, a clear T_{CNT} peak appears. Although its peak energy varies, it is likely due to random strain introduced during the transfer, and we do not see a clear correlation with the WSe_2 layer number. It is noted that in the 1L sample, the PL ratio between T_{CNT} and E_{11} is relatively small, but this does not indicate a weak trion emission. Indeed, T_{CNT} peak is particularly strong as indicated in the PL spectrum on a logarithmic scale in Fig. 3h.

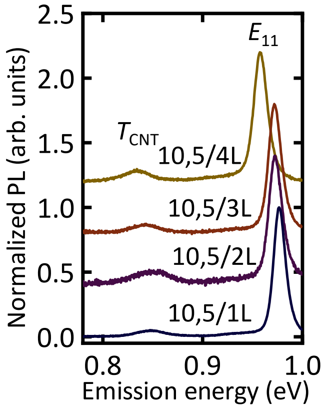

Fig. S2 | PL spectra from the heterostructures with varying WSe_2 thickness. PL spectra of the (10,5) CNT/1, 2, 3, 4L WSe_2 heterostructures. The excitation power is 10 μW and the excitation is at the X_{WSe2}/T_{WSe2} energy of 1.653 eV.

Supplementary Note 3:
Band alignment effect on trion transfer process

Trion transfer strongly depends on the band alignment in the heterostructures. Fig. S3 compares PLE maps from two different samples. In the (14,0) CNT/4L WSe_2 heterostructure, which is known to form a type-II alignment[1], the exciton-transfer feature that produces E_{11} emission at the X_{WSe2}/T_{WSe2} excitation energy (~1.65 eV) is absent. Notably, T_{CNT} emission is also absent across the entire excitation range. By contrast, in the (10,5) CNT/1L WSe_2 heterostructure, which forms type-I band alignment, there is a strong excitation resonance at ~1.65 eV for both E_{11} and T_{CNT} peaks. This observation indicates that type-I band alignment is also curial for trion transfer. Since exciton transfer is suggested to occur through a direct tunneling mechanism[1], type-I alignment provides a small tunneling barrier for both electrons and holes, indicating an efficient transfer. Transferring a trion likely follows a similar process, explaining the observed correlation between exciton and trion transfer processes.

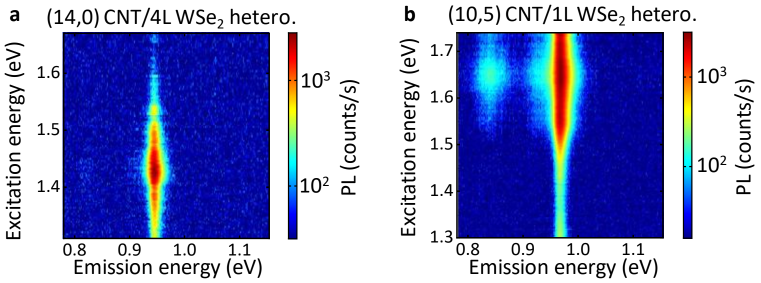

Fig. S3 | PLE maps from different heterostructures. PLE maps of the (14,0) CNT/4L WSe_2 (**a**) and the (10,5) CNT/1L WSe_2 (**b**) heterostructures. The excitation power is 10 μW.

Supplementary Note 4:
Trion transfer dynamics

To characterize the dynamics for both exciton and trion transfer process, we carry out time-resolved PL measurements on three different structures. A (10,5) CNT suspended field-effect transistor structure, which is also shown in Fig. 3c, is first studied to compare the exciton and trion relaxation paths under direct E_{22} excitation condition. Since the relaxation from E_{22} to either E_{11} or T_{CNT} occurs on the order of femtoseconds, the measured decay reveals their lifetimes. The T_{CNT} trion signal is collected under a gate bias V_g of 1.0 V, while the E_{11} exciton signal is collected with V_g of 0 V.

Rapid decay curves are observed for both excitons and trions. We extract the decay lifetime by using an exponential fitting convoluted with the instrument response function (IRF). Two decay components are obtained for the E_{11} PL decay curve (Fig. S4a): A main fast component with a lifetime of 61 ps associated with the bright states, and a small slow component with a lifetime of 640 ps associated with the dark states. In contrast, only one faster decay component of 26 ps appears for the trion T_{CNT} (Fig. S4b). Although the suspended CNT has a different dielectric environment compared to the suspended CNT/WSe_2 heterostructure, it could minimally affect the exciton lifetime, as E_{11} bright exciton lifetime for the suspended CNTs[2] is comparable with that from the heterostructure[1]. We therefore assume that CNT trion lifetime is not changed in different samples.

The intrinsic A exciton X_{WSe2} and the trion T_{WSe2} in a suspended 2L WSe_2 flake are then investigated by exciting at a laser energy of 2.33 eV. Since the PL signals from X_{WSe2} and T_{WSe2} are spectrally unresolvable at room temperature, we collect them together in the time-resolved PL measurement. One slow decay curve is observed in Fig. S4c, indicating that the exciton and the trion has a similar lifetime of 521 ps. The longer lifetime corresponds to the weaker confinement in the 2D system compared to the 1D system.

The longer lifetime in 2D materials leads to a notable reservoir effect. We perform time-resolved PL measurements on the (13,2) CNT/2L WSe_2 heterostructure shown in Fig. 1d, excited at the X_{WSe2}/T_{WSe2} energy. The decay curves for the E_{11} and the T_{CNT} are shown in Fig. S4d and e, respectively. Compared to the lifetimes in Fig. S4a and b, significant slow decay curves with decay times of 334 ps and 281 ps are observed for the exciton and the trion, respectively. The extended decays indicate that excitons and trions generated in WSe_2 continuously transfer into the CNT during their lifetimes, producing the pronounced reservoir effect.

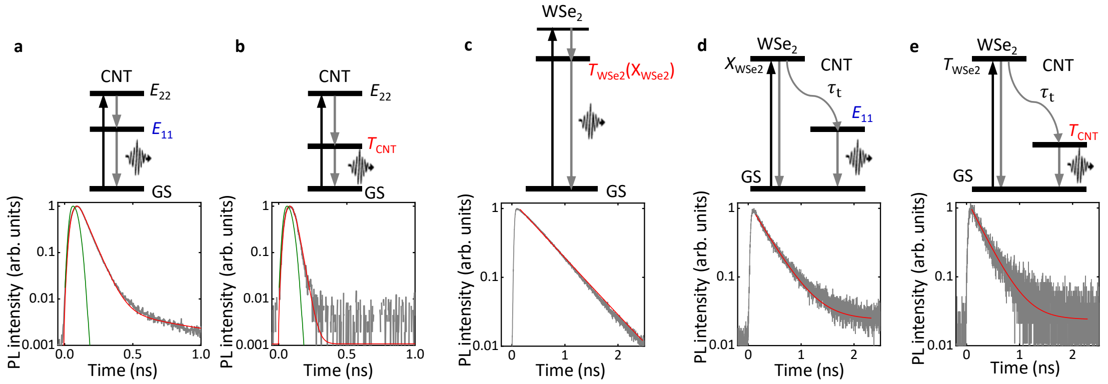

Fig. S4 | Time-resolved PL measurements to estimate trion and exciton transfer ratio. **a, b** PL decay curves from the suspended (10,5) CNT with the field-effect transistor structure. The excitation energy is at E_{22}, and the PL emissions from E_{11} (**a**) and T_{CNT} (**b**) are collected, respectively. **c** A PL decay curve from the suspended 2L WSe_2 flake. The excitation energy is at 2.32 eV, and the PL emission from the WSe_2 flake is collected. **d, e** PL decay curves from the suspended (13,2) CNT/2L WSe_2 heterostructure. The excitation energy is at $X_{\mathrm{WSe2}}/T_{\mathrm{WSe2}}$, and the PL emissions from E_{11} (**d**) and T_{CNT} (**e**) are collected, respectively. The energy level diagrams above each decay curve show the corresponding exciton and trion dynamics. GS indicates the ground state. τ_t represents the transfer time. The excitation power is 2 nW for (**a**, **b**), 100 nW for (**c**), and 4 nW for (**d**,**e**). Long pass filters are used in **b** and **e** to only collect trion PL signals. Experimental results, IRF, and fits are indicated by gray, green, and red lines.

Supplementary Note 5:
Monte Carlo simulation of the transfer process

To gain deeper insight into the trion transfer process and the associated reservoir effect, we perform Monte Carlo simulations that model the trion population profile along one dimension perpendicular to the CNT. The simulation accounts for WSe_2 trion generation, diffusion, decay, and transfer.

Trions in WSe_2 are generated following a Gaussian laser excitation profile with a $1/e^2$ diameter of 1.16 μm, centered on the CNT. During each time interval Δt, trions are allowed to decay with a probability determined by the WSe_2 trion lifetime, and simultaneously diffuse. We let all existing excitons to diffuse with a probability given by the normal distribution $\frac{1}{\sqrt{4\pi D\Delta t}}\exp\left(-\frac{s^2}{4D\Delta t}\right)$, where D is the diffusion coefficient, estimated from a trion lifetime of 521 ps and a diffusion length of 150 nm, and s is the displacement.

Trion transfer occurs probabilistically when a diffusing trion reaches the CNT/WSe_2 heterostructure interface, defined by a boundary width of 1 nm (the CNT diameter). The probability of transfer per interval is $\Delta t/\tau_t$, where τ_t represents the transfer time. To ensure simulation accuracy, Δt is chosen such that the step size is much smaller than the CNT diameter. Once transferred, trions experience an additional residence time, randomly drawn from an exponential distribution determined by τ_t and the CNT trion lifetime (26 ps).

The simulated lifetime distribution of transferred trions (Fig. S5a) reproduces the experimental decay curve (Fig. S4e), yielding a fitted decay lifetime of 280 ps. In this simulation, τ_t = 1.3 ps and the corresponding trion transfer ratio is 20%. The shorter lifetime of transferred trions compared to untransferred ones (Fig. S5b) confirms that many transfer events occur shortly after excitation. The spatial distribution of initially excited trions that eventually transfer into the CNT is shown in Fig. S5c. Compared to the overall excitation profile (Fig. S5d), the transferred trion distribution is narrowly confined, reflecting the limited diffusion length (150 nm) relative to the ~1 μm laser spot size.

We also perform analogous simulations for excitons, using a diffusion length of 1000 nm and a lifetime of 521 ps. A transfer time τ_t = 10 ps and an exciton transfer ratio of 2.0% reproduce the experimental exciton decay (Fig. S4d), resulting in a simulated lifetime of 330 ps (Fig. S5e). Similar to the trions, many excitons transfer shortly after excitation, as evidenced by the longer decay of untransferred excitons (Fig. S5f). Meanwhile, the much longer exciton diffusion length leads to a broader spatial distribution of transferred excitons (Fig. S5g), largely determined by the laser excitation profile (Fig. S5h).

Both exciton and trion transfer exhibit rapid transfer times on the order of 1–10 ps, with substantial transfer ratios considering the dimensional mismatch between 2D and 1D systems. Importantly, trion transfer is more efficient than exciton transfer, resulting in a stronger reservoir effect. The trion emission from the CNT acceptor is therefore

pronounced, while the trion emission from the WSe_2 donor is much weaker compared to exciton emission.

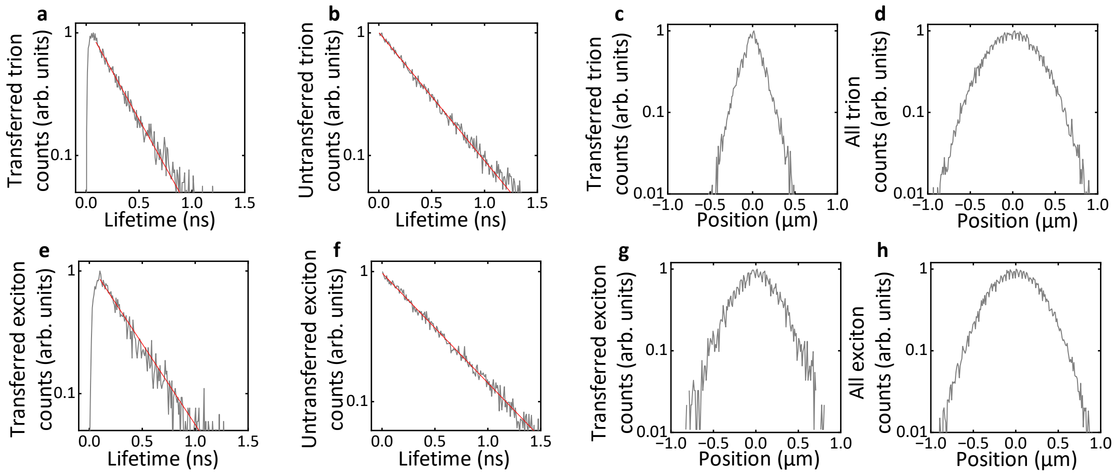

Fig. S5 | Simulated trion and exciton transfer processes. a, b Lifetime distribution of transferred (**a**) and untransferred trions (**b**). **c**, **d** Initial excitation position distribution of transferred (**c**) and all trions (**d**). **e, f** Lifetime distribution of transferred (**e**) and untransferred excitons (**f**). **g**, **h** Initial excitation position distribution of transferred (**g**) and all excitons (**h**). Simulated results and decay fits are indicated by gray and red lines, respectively. The decay lifetimes in **a**, **b**, **e**, **f** are 280, 415, 330, 510 ps, respectively.

Supplementary Note 6:
Excitation polarization characteristics of exciton and trion transfer processes

Excitation polarization measurements offer insights into the dimensional nature of various excited states. We perform excitation-polarization-dependent photoluminescence measurements on the (13,2) CNT/2L WSe_2 heterostructure in Fig. 1d to examine the exciton and trion transfer processes. Under the X_{WSe2}/T_{WSe2} excitation energy, the resulting E_{11} and T_{CNT} emission peaks exhibit no clear linear excitation polarization dependence (Fig. S6a, b). This observation reflects the isotropic, two-dimensional nature of the initial excitons and trions in WSe_2. In contrast, direct excitation at E_{22} yields E_{11} emission with pronounced linear excitation polarization dependence (Fig. S6c), consistent with the expected one-dimensional nature of CNT excitonic states.

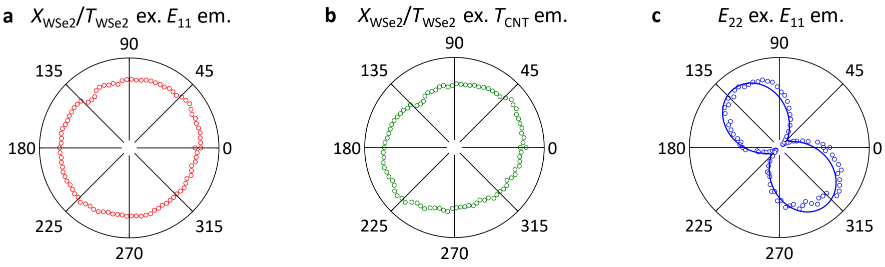

Fig. S6 | Excitation polarization measurements on the heterostructure. Excitation polarization dependence for the (13,2) CNT/2L WSe_2 heterostructure. Red circles indicate X_{WSe2}/T_{WSe2} excitation (1.642 eV) and E_{11} emission (**a**), green circles indicate X_{WSe2}/T_{WSe2} excitation (1.642 eV) and T_{CNT} emission (**b**), and blue circles indicate E_{22} excitation (1.425 eV) and E_{11} emission (**c**), respectively. PL emission is plotted as a function of angle with respect to the trench, where 0 degrees correspond to the direction along the trench. The excitation power is 10 μW. The blue line in (**c**) is a fit to a cosine squared function.

Supplementary Note 7:
Collection efficiency normalization in T_{CNT} efficiency comparison

In Fig. 3i, we compare the PL efficiency of three different structures measured in systems using objective lenses with different numerical apertures (NA). Specifically, NA = 0.65 for the CNT/WSe_2 and suspended gated CNT samples, and NA = 0.80 for the CNT/CuPc samples. Because a higher NA collects light over a larger solid angle, the measured PL signal for the same emission strength can be higher. Thus, to make a fair comparison, we normalize the emission efficiency according to the collection efficiency for each NA.

We model the CNT as a classical electric dipole oriented along the tube axis (the x-axis). In spherical coordinates (θ,ϕ) with θ=0 along the $+z$ axis (the optical axis) and ϕ=0 along the $+x$ axis, the far-field intensity for a linear dipole along x is given by:

$$I\ (\theta,\phi) \propto 1 - \sin^2\theta\cos^2\phi. \quad (1)$$

Here, θ is the polar angle measured from the $+z$ axis, and ϕ is the azimuthal angle in the x–y plane. The differential power radiated into solid angle $d\Omega=\sin\theta d\theta d\phi$ is $dP \propto I(\theta,\phi)\sin\theta d\theta d\phi$. The total power P_{total} emitted by this dipole is obtained by integrating over all solid angles ($0 \le \theta \le \pi$, $0 \le \phi \le 2\pi$):

$$P_{total} \propto \int_{\phi=0}^{2\pi}\int_{\theta=0}^{\pi}[1 - \sin^2\theta\cos^2\phi]sin\theta\ d\theta\ d\phi. \quad (2)$$

An objective of numerical aperture NA in air collects photons emitted within a half-angle θ_{max} defined by $\sin\theta_{max}$=NA. Thus, the collected power is given by integration over $0 \le \theta \le \theta_{max}$, and the collection efficiency (η) is defined as:

$$\eta = \frac{\int_{\phi=0}^{2\pi}\int_{\theta=0}^{\theta max}(1-\sin^2\theta\cos^2\phi)sin\theta\ d\theta\ d\phi}{\int_{\phi=0}^{2\pi}\int_{\theta=0}^{\pi}(1-\sin^2\theta\cos^2\phi)sin\theta\ d\theta\ d\phi}. \quad (3)$$

A numerical integration of Eq. (3) yields a collection efficiency of approximately 16.3% for NA = 0.65 (CNT/WSe_2 and suspended gated CNT samples) and 24.8% for NA = 0.80 (CNT/CuPc samples). To compare all samples on the same effective collection efficiency, we multiply T_{CNT} efficiency and E_{11} efficiency from CNT/CuPc samples by the ratio 16.3%/24.8% ≈ 0.657.

Supplementary Note 8:
Robustness of transfer trion at high carrier densities

We fabricate another gated (10,5) CNT/3L WSe_2 sample and the corresponding gate-dependent PL spectra with V_g rangeing from −10 V (hole doping) to 0 V (near neutral) are shown in Fig. S7a. It is noted that broad background PL emission around 1.1 eV is from the Si substrate. E_{11} is quenched while T_{CNT} remains unaffected as indicated by the peak area versus V_g plots in Fig. S7b, consistent with the data in Fig. 4. We further apply V_g to −20 V, and the resulting PL spectrum (Fig. S7c) shows a strong E_{11} quenching by ~94 %, whereas T_{CNT} persists with little change. This observation highlights the robustness of the trion transfer mechanism, which remains efficient even at elevated free-carrier densities.

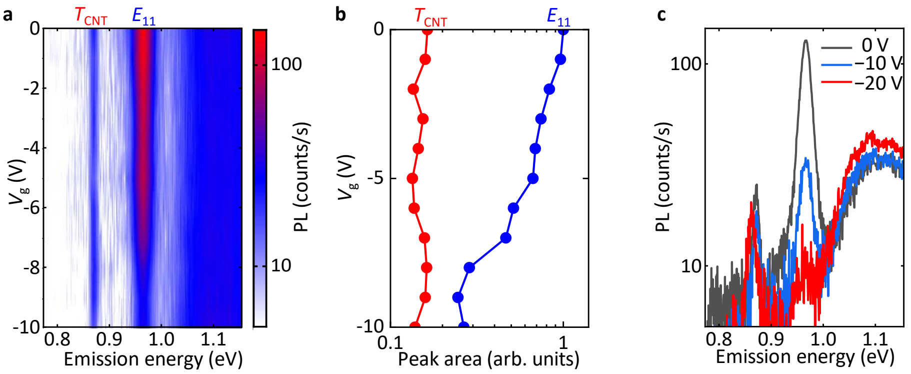

Fig. S7 | Trion transfer in another gated CNT/WSe_2 heterostructure. a PL spectra as a function of gate voltage. The excitation power is 10 μW and the excitation is at the X_{WSe2}/T_{WSe2} energy of 1.642 eV. **b** PL peak area for T_{CNT} (red) and E_{11} (blue) as a function of gate voltage. The peak area is obtained by performing Lorentzian peak fitting on each spectrum in **a** at different V_g. **c** Comparison of PL spectra at V_g = 0, −10, and −20 V.

Supplementary Note 9:
Reduced spatial reservoir effect in CNT/Nb-doped WSe_2 heterostructures

We find a diminished spatial reservoir effect in CNT/Nb-doped WSe_2 heterostructures. PL excitation imaging of a (9,7) CNT/2L doped WSe_2 sample is shown in Fig. S8a–c. Under E_{22} excitation, the E_{11} emission profile follows the suspended CNT shape. When excited at $X_{\mathrm{WSe2}}/T_{\mathrm{WSe2}}$, the E_{11} emission is only slightly broadened. Under the same excitation energy, the T_{CNT} emission exhibits negligible spatial broadening along the trench. The line profiles in Fig. S8d clearly illustrates these differences. Compared to the laser spot profile determined by the E_{11} emission under E_{22} excitation, the small broadening of E_{11} under $X_{\mathrm{WSe2}}/T_{\mathrm{WSe2}}$ excitation yields the exciton diffusion length of 0.12 μm extracted from the fit. Meanwhile, the broadening of the T_{CNT} emission under $X_{\mathrm{WSe2}}/T_{\mathrm{WSe2}}$ excitation is unresolvable, indicating an even smaller trion diffusion length that falling below the resolution of our imaging setup.

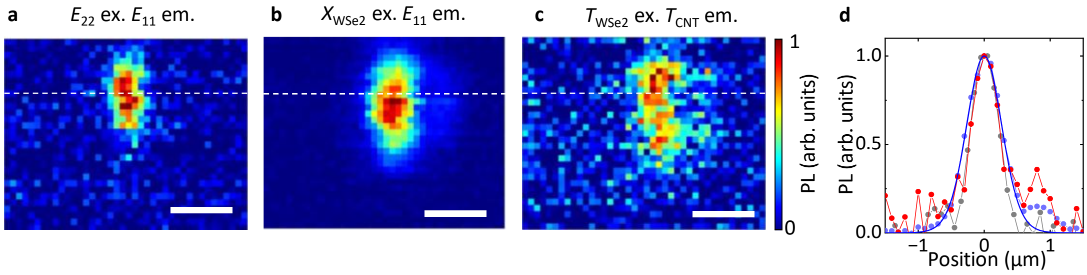

Fig. S8 | PL excitation images for the CNT/doped WSe_2 heterostructure. a-c Normalized PL intensity maps from the (9,7) CNT/2L doped WSe_2 sample. The PL images are constructed by integrating PL emission over a 20-meV-wide spectral window centered at E_{11} energy (**a**, **b**) and T_{CNT} energy (**c**). The excitation is at E_{22} (1.540 eV, a), X_{WSe2} (1.664 eV, b), and T_{WSe2} (1.664 eV, c). The excitation power is 10 μW. The scale bars represent 1 μm. **d** Line profiles taken from **a-c**, as indicated by white broken lines. The grey, blue, and red symbol-line plots are the experimental results from **a**, **b**, and **c**, respectively. The blue line is the fit for the results from **b**.

References

(1) Fang, N.; Chang, Y.-R.; Yamashita, D.; Fujii, S.; Maruyama, M.; Gao, Y.; Fong, C. F.; Otsuka, K.; Nagashio, K.; Okada, S.; Y. K. Kato, Resonant exciton transfer in mixed-dimensional heterostructures for overcoming dimensional restrictions in optical processes. *Nat. Commun.*, **2023**, *14*, 8152.

(2) Ishii, A., Machiya, H.; Y. K. Kato, High efficiency dark-to-bright exciton conversion in carbon nanotubes. *Phys. Rev. X*, **2019**, *9*, 041048.
